# Pain, duration and safety of computer-assisted surgical exposure of palatally displaced canines: A case series

**DOI:** 10.4317/medoral.27530

**Published:** 2025-10-14

**Authors:** María Lara-Muros, Cristina de-la-Rosa-Gay, Javi Vilarrasa, Berta García-Mira, Rui Figueiredo, Eduard Valmaseda-Castellón, Octavi Camps-Font

**Affiliations:** 1 PhD student. Faculty of Medicine and Health Sciences of the University of Barcelona, Barcelona, Spain; 2 Department of Dentistry, Faculty of Medicine and Health Sciences of the University of Barcelona, Barcelona, Spain; 3 Dental and Maxillofacial Pathology and Therapeutics Research Group. IDIBELL Research Institute, Barcelona, Spain; 4 Department of Periodontology, International University of Catalonia, Barcelona, Spain; 5 Department of Oral Surgery and Implantology, University of Valencia, Valencia, Spain

## Abstract

**Background:**

Surgical guides have recently been introduced in application to the open exposure of palatally displaced canines (PDCs). The present study assesses postoperative pain, fitting of the guide, surgery time and safety of the procedure.

**Material and Methods:**

A prospective case-series was conducted from March 2023 to October 2024. Patients 12 to 20 years of age with at least one PDC requiring treatment with a combined orthodontic fixed appliances and surgical approach were included. Surgical templates were obtained after virtual planning. An intraoral scan was superimposed with cone-beam computed tomography to design the guide with a window according to the canine position. Flapless open exposure using the guide was performed, employing a scalpel and ostectomy with burs if needed. Surgery time (from the administration of local anesthesia to the start of the orthodontic attachment bonding or the placement of the protective pack), guide adjustment and intra-surgical complications were also reported. A questionnaire was given to the patient to record postoperative pain, analgesic consumption and any possible adverse event. Descriptive and bivariate analyses were performed.

**Results:**

Ten patients (14 PDCs) were included. Computer-assisted PDC exposure lasted a median of 26 minutes (IQR = 18.00), and no complications were reported. All patients experienced mild post-operative pain (i.e., VAS &lt; 40 mm). Pain intensity peaked between 2 and 24 hours post-surgery and gradually decreased over time. Surgical guides successfully fit in all cases. No fitting issues were noted that affected the accurate placement or functionality of the guide.

**Conclusions:**

Computer-guided exposure of PDCs is a feasible minimally invasive approach that reduces surgery time and postoperative pain. The use of an individualized guide is an easy tool for increasing the safety and efficacy of this procedure.

## Introduction

Permanent upper canines usually erupt at 11 to 13 years of age. Displacement or impaction of these teeth is relatively common. Palatally displaced canines (PDCs) are present in 2-3% of young individuals, which makes this the second most frequent dental impaction after third molars (1 , 2). PDCs represent approximately two-thirds of all impacted upper canines, with 8-10% of the cases being bilateral (3).

The etiology of PDC is multifactorial, involving both genetic and acquired factors. In effect, while some studies suggest a genetic background (4), local factors may also contribute (5). In particular, lateral incisor hypodontia and microdontia have been associated with canine impaction (6).

Canine impaction requires timely and effective treatment. Currently, the most widely adopted approach to PDC treatment is a combination of surgical exposure and orthodontic treatment. Two main traction techniques have been described: open and closed. In the open technique, the overlying mucosa is removed, so that the tooth is visible during traction, while in the closed technique the palatal mucosa is repositioned and pierced by the traction device. Both approaches have demonstrated similar outcomes in terms of safety, success rates, treatment duration, periodontal health, and patient-reported satisfaction (1 , 5 , 7 - 9). Thus, the choice of technique is typically determined by clinicians based on the specific case and their clinical preference (1).

Guided surgery has been introduced in dentistry with the aim of increasing accuracy and safety, as well as reducing surgery time, invasiveness and morbidity. Several studies have demonstrated the effectiveness of guided surgery in implantology or periodontics (10 , 11), with reduced morbidity and improved accuracy. Despite these clinical advantages, guided surgery requires an increase in preoperative planning time and additional costs related to specialized software and hardware, instruments or the guide manufacture. A learning curve is still necessary.

Recently, guided surgery, either static (12 , 13) or dynamic (14), has been used for the extraction, transplantation of impacted teeth or surgical exposure of PDC (15 , 16).

Static guided surgery uses surgical templates to transfer a digital plan into the oral cavity. To start planning, a cone-beam computed tomography (CBCT) scan and a digital impression are needed. These two 3D images are then superimposed, and a customized surgical template is manufactured. A recent randomized clinical trial has compared free-hand and computer-assisted exposure of PDCs (16). Surgery time was significantly reduced, and no complications were reported. However, evidence in the guided open exposure of PDCs is still scarce (15).

The main aim of the present study was to assess postoperative pain in a case series of computer-assisted exposures of PDCs using a surgical template. The secondary objective was to analyze fitting of the surgical template, surgery time, and the safety of the procedure.

## Material and Methods

A prospective case-series study was conducted in 10 outpatients consecutively treated (March 2023 to October 2024) in a private dental clinic (Espai Dental la Garriga, Barcelona, Spain). The study design followed the STROBE guidelines for observational studies (17). The protocol was developed in accordance with the Declaration of Helsinki (18) and was approved by the Ethics Committee (CEIm) of the Dental Hospital of the University of Barcelona (Barcelona, Spain) (Protocol number 13/2024).

Patients received comprehensive information about the surgical procedures and treatment alternatives, and informed consent was obtained in all cases. In patients under the age of 18, consent was also obtained from the legal guardian.

Patient selection

The inclusion criteria were: patients aged 12 to 20 years with at least one PDC requiring treatment with a combined fixed orthodontic appliances and surgical approach; patients with an American Society of Anesthesiologists (ASA) physical status classification score of I (19); dentoalveolar development stage classified as DS2M1 or DS4M3 according to Björk et al. (20); and full mental capacity to understand the study and provide informed consent before participation, as well as willingness to comply with all the scheduled visits and procedures as outlined in the study protocol. At the time of the surgery, all patients had been wearing fixed appliances, such as full upper-arch braces or a palatal bar, for at least one month. Only one patient had a miniscrew in the palate.

The exclusion criteria were: general contraindications to oral surgery, such as radiotherapy of the head and neck region, uncontrolled diabetes, or alcohol abuse; craniofacial deformities including cleft lip or palate; a history of any disease or condition which in the opinion of the researcher could pose a risk to the patient or potentially affect the efficacy and safety results of the study; current pregnancy or lactation; and inclusion in other clinical studies in the previous weeks. An additional exclusion criterion was an impacted maxillary canine complexity KPG index 20 (extremely difficult) (21).

Surgical guide design and manufacturing

Digital Imaging and Communication in Medicine (DICOM) files were obtained using a CBCT scan (NewTom GiANO HR; Quantitative Radiology, Verona, Italy; resolution: 0.2 mm/voxel, 110 kV, 64.6 mAs, 4.3 s). Standard Tessellation Language (STL) files were acquired with an intraoral scanner (Trios 3; 3Shape, Copenhagen, Denmark). Both images were superimposed by one researcher (O.C.F.) using Exocad DentalCAD software (Exocad GmbH, Darmstadt, Germany). A surgical guide was virtually planned to cover the incisal and occlusal surfaces up to the first upper molars and the palate, leaving inspection windows to check the correct fitting of the guide in the upper arch. A window was designed on the crown of the PDC for the open exposure, preserving at least 3 mm of gingiva in the neighboring teeth. The offset to set the clearance between the guide and the patient's contact surface was 0.03 mm, and the wall thickness of the surgical guide was 2.5 mm. Once the virtual design of the surgical guide was completed, the data was exported to a 3D printer with selective laser sintering (SLS) technology and a layer thickness of 60 to 120 µm (Formiga P110; EOS - Electro Optical Systems, Krailing, Germany). The post-processing of the surgical guide included sandblasting with glass microbeads, quality control, cleaning in ultrasonic baths, and cleaning with a thermo-disinfection machine. Finally, the surgical guide was sterilized in an autoclave oven at 134ºC with a pressure of 2 bar.

Surgical protocol

1

**Figure 1:**
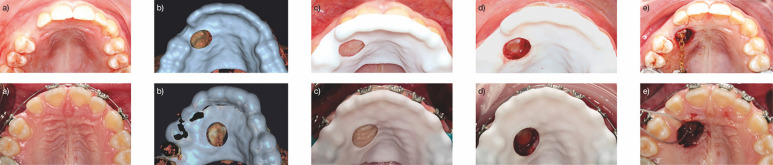
Surgical views in two clinical cases. (a) Preoperative view of the upper arch. (b) Virtual design of the guide. (c) Guide in placeprior to exposure. (d) Guide in position after ostectomy. (e) Bonded device in place.

After surgery, ibuprofen 400 or 600 mg p.o. 3 times a day for 4 to 5 days was prescribed. Paracetamol 650 mg p.o. 3 times per day was prescribed as rescue medication. Patients were instructed to rinse with 15 ml of 0.12% chlorhexidine digluconate (PerioAid Tratamiento 0.12% (Dentaid, Cerdanyola del Vallès, Spain) twice a day for 15 days. Both oral and written postoperative instructions, including detailed recommendations, were provided to the patients. They were also given a questionnaire to report postoperative pain intensity for the next 7 days, the occurrence of any adverse events and analgesic consumption. A follow-up appointment was scheduled 7 days after the surgical procedure to monitor healing and submit the questionnaire.

During the first week after the procedure, no traction nor dental movement was initiated to avoid interference with pain assessment.

Data sampling

The primary outcome was patient-reported pain, which was recorded at the end of the surgical procedure, then at 2, 6, 12 and 24 hours, and daily until the 7th postoperative day using a 100-mm visual analogue scale (VAS), where 0 = 'no pain' and 100 = 'worst pain imaginable'.

The registered secondary outcomes were:

1. Intra-surgical complications.

2. Any adverse medical event, unexpected illness or injury, or undesired clinical signs associated with the open PDC surgical procedure.

3. Surgery duration, recorded in minutes, from the administration of local anesthesia to the start of the orthodontic attachment bonding or placement of the protective pack.

4. Clinician experience with the PDC surgical procedure and adjustment of the surgical guide evaluated using a Likert scale.

5. Analgesic consumption (ibuprofen or paracetamol) during the first 7 postoperative days.

Sample size calculation

The sample size was calculated using G*Power software v.3.1.3 (Heinrich-Heine Universität, Düsseldorf, Germany), based on the assumption that pain intensity would vary significantly over time. With a partial ² of 0.1 mm, a significance level () of 0.05, a statistical power (1-) of 90%, and 11 measurement timepoints, 10 patients were seen to be needed.

Statistical analysis

Statistical analyses were performed using STATA 14.1 software (StataCorp®, College Station, TX, USA). Categorical variables were summarized as absolute and relative frequencies. Results were reported as the median and interquartile range (IQR). Correlations between scale variables were tested with Spearman's rank order correlation coefficient. To evaluate the effect of the procedure on pain evolution over time, longitudinal data were analyzed using the Friedman test. Statistical significance was set at p&lt;0.05 for all analyses.

## Results

Ten patients (14 PDCs) with a median age of 13.5 years (IQR: 1.66) were included in the study. All participants completed the treatment protocol without deviations or dropouts. They were all classified as ASA I, with no systemic diseases. According to the modified KPG index, 3 canines (21.43%, 2 patients) were classified as difficult (i.e., scores between 15 and 19), 8 cases (57.14%, 6 patients) as moderately difficult (i.e., scores between 7 and 14), and the remaining 3 cases (21.43%, 2 patients) as easy (i.e., scores 7). The main demographic and clinical characteristics of the study sample are presented in Table 1.

Table 1

**Figure 2:**
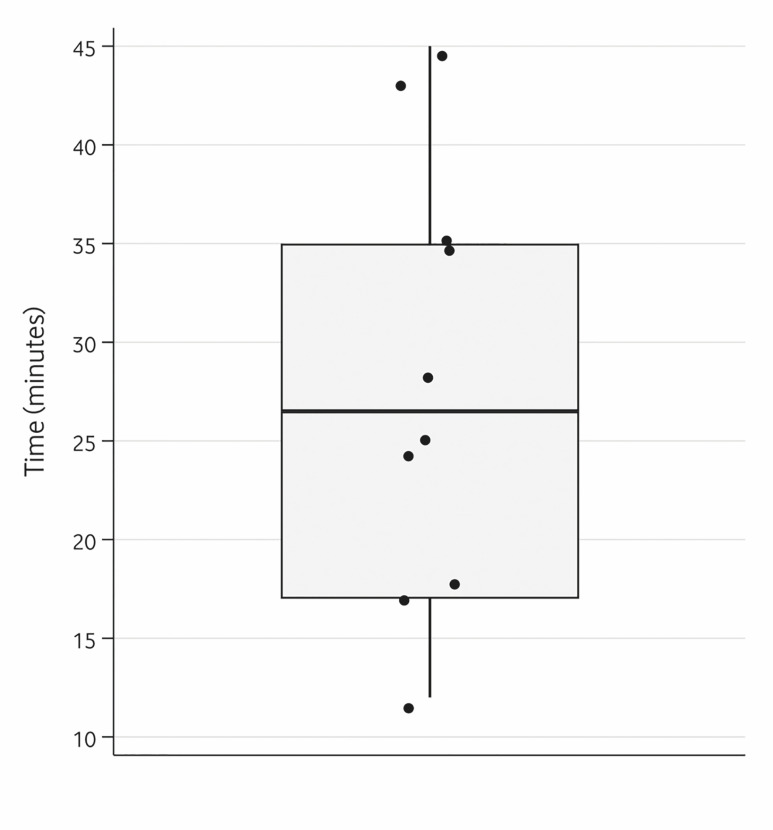
Boxplot of postoperative time. The points indicate individualvalues.

Guided PDC surgery lasted a median of 26.5 minutes (IQR=18.00) from anesthesia to the start of orthodontic attachment bonding or placement of the protective pack (Figure 2). A surgical cement (COE-PAKTM, GC Corporation, Tokyo, Japan) was placed in three cases because the Orthodontist preferred to bond the traction device. All canines required some degree of ostectomy. Surgery time was positively correlated with the treatment difficulty score (=0.66; p=0.041). No intraoperative or postoperative complications were recorded.

2

Furthermore, the surgeon rated fitting of the surgical guide as excellent in 6 cases (fit and desired exposure of canine) and very good (fit but with the opening slightly larger than the canine) in 4 patients. In any case, it allowed successful exposure of the PDC. No guide fractures or gaps were observed.

**Figure 3:**
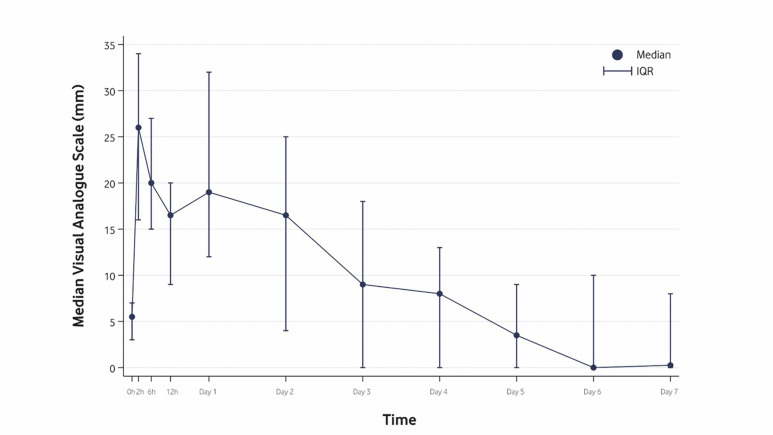
Median of the visual analogue scale with IQR (interquartile range). The horizontal axis representstime after surgery, while the vertical axis describes patient-reported (VAS) pain (from 0 to 100mm). Thepain scale has been truncated at 35mm.

**Figure 4:**
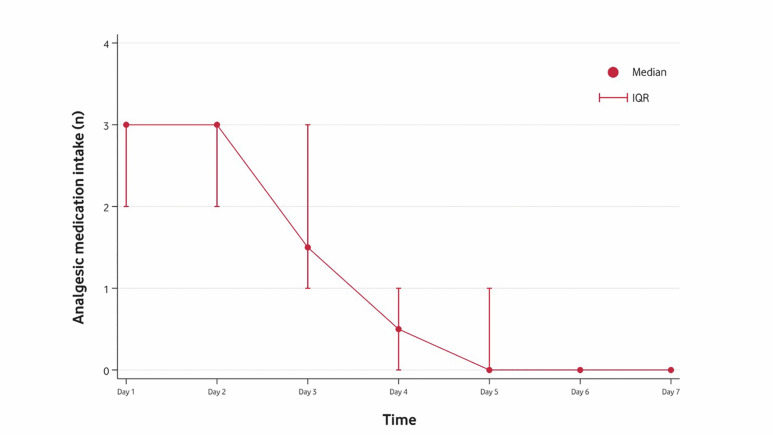
Median of the number of analgesic tablets consumed per day with IQR (interquartile range). Thehorizontal axis represents postoperative days, while the vertical axis measures the number of tablets per day.

The postoperative VAS pain scores varied significantly over time (Q=80.69; df=10; p&lt;0.001). All patients experienced mild pain (i.e., VAS &lt;40mm) throughout the entire follow-up period. The peak of pain occurred between 2 to 24 hours post-intervention and then progressively decreased over time (Figure 3). Analgesic consumption followed a similar pattern (Q=49.05; df=6; p&lt;0.001) (Figure 4).

3

4

## Discussion

Computer-assisted surgical exposure of PDC has proved to be a safe and predictable approach (16). In the present prospective case-series study, surgeries lasted less than half an hour, patients experienced mild pain, and no complications were reported.

Surgery time is crucial when evaluating the efficiency of any technique. In our study, the mean surgical time was 26.5 minutes (IQR=18.00) from anesthesia to the start of orthodontic attachment bonding or placement of the protective pack. In conventional open exposures, three previous studies have reported mean surgery times from initial incision to last suture of 30.9 minutes (SD=10.1) (22), 34.3 minutes (SD=11.2) (23) and 28 minutes (SD=12.9) (8), respectively. Our findings suggest a potential reduction in surgery time when using a guide. This is especially meaningful in the young population, as treatment duration affects patient behavior (24). Besides, longer surgery times have been associated with a higher risk of postoperative complications (25).

A recent randomized clinical trial (16) compared guided versus conventional surgical exposure of PDCs. In the guided surgery group, the mean surgery time was 4 minutes and 45 seconds (SD=68.4 seconds) using computer-assisted surgery, and 7 minutes and 22.3 seconds (SD=56.0 seconds) with the conventional approach. In the present case series, the surgery time using computer-assisted surgery was longer; however, the surgery times reported in other studies on conventional open exposure are generally longer than those observed in the control group of this study (8 , 22 , 23). Several factors may account for these discrepancies in surgery time, such as variations in the definition of surgery time, differences in surgical technique, and heterogeneity in case complexity. In this regard, Kivovics et al. (16) measured surgery time from the onset of the procedure, whereas in the present study, timing was initiated at the administration of local anesthesia. Additionally, the mentioned authors employed a laser for gingivectomy, which may have provided superior hemostasis and operative efficiency compared to the scalpel used in our cases. Moreover, the level of surgical difficulty was not reported in their study; notably, not all cases required ostectomy, in contrast to our series, where ostectomy was performed in all cases. Computer-assisted exposure of PDCs delineates the surgical field better and reduces invasiveness, in a way similar to flapless guided implant surgery (26).

Surgery time is also used to estimate surgical difficulty (27). Accordingly, we observed a correlation between surgery time and case complexity. Canines with a higher KPG index required significantly more time for exposure. Usually, the higher the KPG index, the greater the need for ostectomy and thus, the risk of iatrogenic damage to adjacent teeth. When using a surgical template, the surgical area is clearly delimited by a window, and there is no need for frequently checking the CBCT scan and the adjacent structures. In consequence, procedures are safer and more predictable, thus reducing chair time, particularly in PDCs with a high KPG index.

PDC open exposure is a relatively straightforward procedure. We did not record any intra- or immediate postoperative complications. Among the possible complications are intraoral bleeding, wound dehiscence, infection or injury to the adjacent teeth or anatomical structures (1). The palatal mucosa is highly vascularized, particularly in canines close to the midline, and the nasopalatine vessels or the incisor roots may be injured. The improvement of accuracy and safety provided by surgical guides might reduce the likelihood of complications, as seen in implant placement (26 , 28).

Pain is a multifactorial experience. It is affected by patient gender, age, cultural and educational aspects, extension of the procedure (i.e., bilateral impaction) (9), and case complexity, among other factors. In our study, all patients reported mild pain after the computer-assisted exposure of PDCs and the 7 days of follow-up (VAS&lt;40mm). In contrast to a previous study (16), in no case did the pain cross the threshold of moderate or severe intensity, with the pain peak appearing within the first 24 hours and subsequently decreasing, as in other studies (22 , 23). Despite pain evolution being similar, our peak values were shorter (24 hours versus 72 hours), reflecting faster recovery (16). Thus, the use of a surgical guide leads to less postoperative pain, probably due to the flapless approach and the selective ostectomy.

CAD/CAM surgical templates are an easy-to-handle guided option. Nowadays, they are also easy to plan and manufacture. A wide range of digital software can be used to design access and plan the procedure. This 3D planning may be particularly useful in cases with deep impaction, incisor resorption or severe crowding, where damage to neighboring teeth becomes more likely. Despite requiring additional off-chair clinician time, it reduces actual chair time and makes surgery more predictable. Template fitting was excellent in 60% of the cases, while in the remaining 40%, it was considered very good. These results highlight the stability and precision of tooth-supported surgical guides.

There are also some limitations in computer-assisted guided surgery. Errors from digital impressions, inaccuracies in the CBCT scan or manufacturing errors can impair fitting of the templates (28). However, we did not have to face these inconveniences, as the template fitting was excellent or very good in all cases. It is also crucial to keep safety distances around adjacent teeth and anatomical structures. This also happens in cases treated with conventional surgery, but with guided approach, the likelihood of iatrogenic damage decreases, and accuracy is improved. Using a surgical template requires a learning process. Nevertheless, in our study, the operator had the required expertise both in PDC exposure and guided surgery. The enhanced accuracy and safety achieved with surgical templates make the additional cost of the guide and software worthwhile. Finally, static computer-assisted guided surgery does not provide intraoperative feedback as dynamic or robotic systems do (14 , 29). Further investigations may address this drawback.

## Conclusions

Our findings suggest that the computer-assisted surgical exposure of PDCs is a promising minimally invasive approach for reducing surgery time and minimizing postoperative pain. CAD/CAM surgical guides for the exposure of PDCs are easy to adapt and increase patient safety. Further research with adequate follow-up is necessary to validate these findings and detect potential long-term complications. The small sample size involved, and the lack of a control group are limitations of our study. Randomized controlled trials comparing this novel technique with the conventional approach are recommended.

## Figures and Tables

**Table 1:** Table Patient age, gender, number of PDCs, difficulty (KPG index) and type of treatment.

Case	Age (years)	Sex	Canine	KPGindex	Treatment*
1	16.89	Female	2	18	Bonding
				17	Bonding
2	14.86	Female	2	13	Bonding
				8	Bonding
3	13.30	Female	1	10	Bonding
4	13.30	Male	2	7	Bonding
				9	Bonding
5	13.15	Male	1	11	Cement
6	13.01	Female	1	19	Bonding
7	14.55	Male	2	5	Bonding
				5	Bonding
8	13.20	Female	1	11	Cement
9	13.63	Female	1	6	Bonding
10	15.55	Female	1	7	Cement

Bonding: Bonding of a traction device at surgery. Cement: Suturing of cement over the PDC exposed crown, without bonding.

## Data Availability

Declared none.

## References

[1] Parkin N, Benson PE, Thind B, Shah A, Khalil I, Ghafoor S (2017). Open versus closed surgical exposure of canine teeth that are displaced in the roof of the mouth. Cochrane Database Syst Rev.

[2] Grover PS, Lorton L (1985). The incidence of unerupted permanent teeth and related clinical cases. Oral Surg Oral Med Oral Pathol.

[3] Bishara SE, Ortho D (1992). Impacted maxillary canines: A review. Am J Orthod Dentofacial Orthop.

[4] Vitria EE, Tofani I, Kusdhany L, Bachtiar EW (2019). Genotyping analysis of the Pax9 Gene in patients with maxillary canine impac-tion. F1000Res.

[5] Camilleri S (2021). Canine exposure: Open or closed?. Eur J Orthod.

[6] Papageorgiou SN, Seehra J, Cobourne MT, Kanavakis G (2025). Does current evidence support the discussion around the guidance theory? A systematic review and meta-analysis on the association between maxillary lateral incisor agenesis and displacement or impaction of the permanent canine. Orthod Craniofac Res.

[7] Sampaziotis D, Tsolakis IA, Bitsanis E, Tsolakis AI (2018). Open versus closed surgical exposure of palatally impacted maxillary canines: comparison of the different treatment outcomes-a systematic review. Eur J Orthod.

[8] Björksved M, Arnrup K, Lindsten R, Magnusson A, Sundell AL, Gustafsson A (2018). Closed vs open surgical exposure of palatally displaced canines: surgery time, postoperative complications, and patients' perceptions: a multicentre, randomized, controlled trial. Eur J Orthod.

[9] Björksved M, Arnrup K, Bazargani SM, Lund H, Magnusson A, Magnuson A (2021). Open vs closed surgi-cal exposure of palatally displaced canines: a comparison of clinical and patient-reported outcomes-a multicentre, randomized controlled trial. Eur J Orthod.

[10] Coachman C, Valavanis K, Silveira FC, Kahn S, Tavares AD, Mahn E (2023). The crown lengthening double guide and the digital Perio analysis. J Esthet Restor Dent.

[11] Jorba-García A, Bara-Casaus JJ, Camps-Font O, Figueiredo R, Valmaseda-Castellón E (2023). Accuracy of dental implant placement with or without the use of a dynamic navigation assisted system: a randomized clinical trial. Clin Oral Implants Res.

[12] Gómez Meda R, Abella Sans F, Esquivel J, Zufía J (2022). Impacted maxillary canine with curved apex: three-dimensional guided protocol for autotransplantation. J Endod.

[13] Abella F, Ribas F, Roig M, González-Sánchez JA, Durán-Sindreu F (2018). Outcome of autotransplantation of mature third molars using 3-dimensional-printed guiding templates and donor tooth replicas. J Endod.

[14] Wang J, Cui NH, Guo YJ, Zhang W (2017). Navigation-guided extraction of impacted supernumerary teeth: a case report. J Oral Maxillofac Surg.

[15] Camps-Font O, Vilarrasa J (2023). Computer-guided surgical exposure of palatally displaced canines: a technical note. Int J Comput Dent.

[16] Kivovics M, Szanyi SM, Takács A, Répási M, Németh O, Mijiritsky E (2024). Computer-assisted open exposure of palatally impacted canines for orthodontic eruption: a randomized clinical trial. J Dent.

[17] von Elm E, Altman DG, Egger M, Pocock SJ, Gøtzsche PC, Vandenbroucke JP (2008). The Strengthening the Reporting of Observa-tional Studies in Epidemiology (STROBE) statement: guidelines for reporting observational studies. J Clin Epidemiol.

[18] Halonen JI, Erhola M, Furman E, Haahtela T, Jousilahti P, Barouki R (2020). The Helsinki Declaration 2020: Europe that pro-tects. Lancet Planet Health.

[19] Hurwitz EE, Simon M, Vinta SR, Zehm CF, Shabot SM, Minhajuddin A (2017). Adding examples to the ASA-physical status classification improves correct assignment to patients. Anesthesiology.

[20] Björk A, Krebs A, Solow B (1964). A method for epidemiological registration of malocclusion. Acta Odontol Scand.

[21] Chung HK, Pan P, Gallerano RL, English JD (2009). A novel 3D classification system for canine impactions-the KPG index. Int J Med Robot.

[22] Gharaibeh TM, Al-Nimri KS (2008). Postoperative pain after surgical exposure of palatally impacted canines: closed-eruption versus open-eruption, a prospective randomized study. Oral Surg Oral Med Oral Pathol Oral Radiol Endod.

[23] Parkin NA, Deery C, Smith AM, Tinsley D, Sandler J, Benson PE (2012). No difference in surgical outcomes between open and closed exposure of palatally displaced maxillary canines. J Oral Maxillofac Surg.

[24] Jamali Z, Najafpour E, Ebrahim Adhami Z, Sighari Deljavan A, Aminabadi NA, Shirazi S (2018). Does the length of dental treatment influence children's behaviour during and after treatment? A systematic review and critical appraisal. J Dent Res Dent Clin Dent Prospects.

[25] Morcos MW, Nowak L, Schemitsch E (2021). Prolonged surgical time increases the odds of complications following total knee arthroplasty. Can J Surg.

[26] Romandini M, Ruales-Carrera E, Sadilina S, Hämmerle CHF, Sanz M (2023). Minimal invasiveness at dental implant placement: a systematic review with meta-analyses on flapless fully guided surgery. Clin Oral Implants Res.

[27] Sánchez-Torres A, Soler-Capdevila J, Ustrell-Barral M, Gay-Escoda C (2020). Patient, radiological, and operative factors associated with surgical difficulty in the extraction of third molars: a systematic review. Int J Oral Maxillofac Surg.

[28] Tatakis DN, Chien HH, Parashis AO (2019). Guided implant surgery risks and their prevention. Periodontol 2000.

[29] Wang Y, Wang Y, Yu S, Han L, Feng Y, Miron RJ, et al. (2024). Influence of dental implant clinical experience on the accuracy of robot-assisted immediate implant placement: an in vitro study. Clin Oral Investig.

